# TMPRSS4 facilitates epithelial-mesenchymal transition of hepatocellular carcinoma and is a predictive marker for poor prognosis of patients after curative resection

**DOI:** 10.1038/srep12366

**Published:** 2015-07-20

**Authors:** Cheng-Hao Wang, Zhong-Yi Guo, Ze-Ting Chen, Xu-Ting Zhi, Deng-Ke Li, Zhao-Ru Dong, Zhi-Qiang Chen, San-Yuan Hu, Tao Li

**Affiliations:** 1 Department of general surgery, Qilu Hospital, Shandong University, Jinan 250012, P.R.China; 2 Liver Cancer Institute and Zhongshan Hospital, Fudan University, Key Laboratory for Carcinogenesis & Cancer Invasion, the Chinese Ministry of Education, Shanghai 200032, P.R.China

## Abstract

TMPRSS4 (Transmembrane protease serine 4) is up-regulated in a broad spectrum of cancers. However, little is known about the biological effects of TMPRSS4 on hepatocellular carcinoma (HCC) and the related mechanisms. In the present study, we found that overexpression of TMPRSS4 significantly promoted the invasion, migration, adhesion and metastasis of HCC. Further more, TMPRSS4 induced EMT of HCC, which was mediated via snail and slug as a result of Raf/MEK/ERK1/2 activation, and inhibition of ERK1/2 activation by its inhibitor was associated with reduced cell invasion and reversion of EMT. In addition, we demonstrated that TMPRSS4 remarkably suppressed the expression of RECK, an inhibitor of angiogenesis, and drastically induced tumor angiogenesis and growth. More important, in clinical HCC specimens, TMPRSS4 expression was significantly correlated with tumor staging and was inversely correlated with E-cadherin and RECKS expression. Expression of TMPRSS4 is significantly associated with HCC progression and is an independent prognostic factor for postoperative worse survival and recurrence. In conclusion, TMPRSS4 functions as a positive regulator of Raf/MEK/ERK1/2 pathway and promotes HCC progression by inducing EMT and angiogenesis. The increase of TMPRSS4 expression may be a key event for HCC progression and may be regarded as a potential prognostic marker for HCC.

Proteases contribute to degradation of the basement membrane and extracellular matrix (ECM) and to tissue remodeling, thereby play important roles in the development and homeostasis of an organism^1^. In addition to that, the importance of proteases as a key player in cancer progression has been increasingly recognized. Aside from their ability to degrade the ECM, facilitate invasion and metastasis, proteases are also signaling molecules that modulate a great variety of other molecules by underlying pathways, form interconnected cascades, circuits and networks that may be involved in all stages of the development and progression of cancer, including growth, migration, invasion, angiogenesis and metastasis^2^.

Recently, much attention has been focused on the role of type II transmembrane serine proteases (TTSPs), which are members of the family of cell surface-associated proteases and have in common a proteolytic domain, a transmembrane domain, a short cytoplasmic domain and a variable-length stem region containing modular structural domains^3^,^4^,^5^,^6^. TTSPs are distinct from other serine proteases in that they have an integral transmembrane domain, while most other serine proteases are secreted. The cytoplasmic tail of TTSPs enables them to interact with cytoskeletal and cellular signaling molecules, mediate a variety of normal cellular functions, regulate cell-cell and cell-matrix interactions as well as tumor invasion and metastasis^6^. Increasing evidence demonstrates that aberrant expression of TTSPs is a hallmark of several cancers and recent studies have defined molecular mechanisms underlying TTSP-promoted carcinogenesis^7^.

TMPRSS4 (Transmembrane protease serine 4) is a novel TTSPs found at the cell surface that is highly expressed in pancreatic, colon, thyroid and gastric cancer tissues^6^. The biological functions of TMPRSS4 during tumorigenesis has recently been reported, and TMPRSS4 has been shown to be an important mediator during invasion, metastasis, migration, adhesion and the epithelial-mesenchymal transition (EMT) in human epithelial cancer cells, and may represent a new therapeutic target for cancers^1^,^8^,^9^. However, the biological functions of TMPRSS4 have not yet been elucidated in hepatocellular carcinoma (HCC). In the present study, we show that TMPRSS4 significantly promoted the invasion, migration, adhesion, metastasis of HCC through inducing EMT, which was mediated via snail and slug as a result of Raf/MEK/ERK1/2 activation. We demonstrate, for the first time, TMPRSS4 remarkably suppresses the expression of RECK, an inhibitor of angiogenesis, drastically induces tumor angiogenesis. Our data also demonstrates that increased expression of TMPRSS4 is significantly associated with HCC progression and a worse patient survival, and is an independent prognostic factor for postoperative recurrence. These findings provide new evidence for the involvement of TMPRSS4 in the connection between EMT and angiogenesis and identify TMPRSS4 as a potential diagnostic and prognostic marker for HCC.

## Results

### TMPRSS4 overexpression is associated with morphological changes and EMT gene regulation

**Figure 1:**
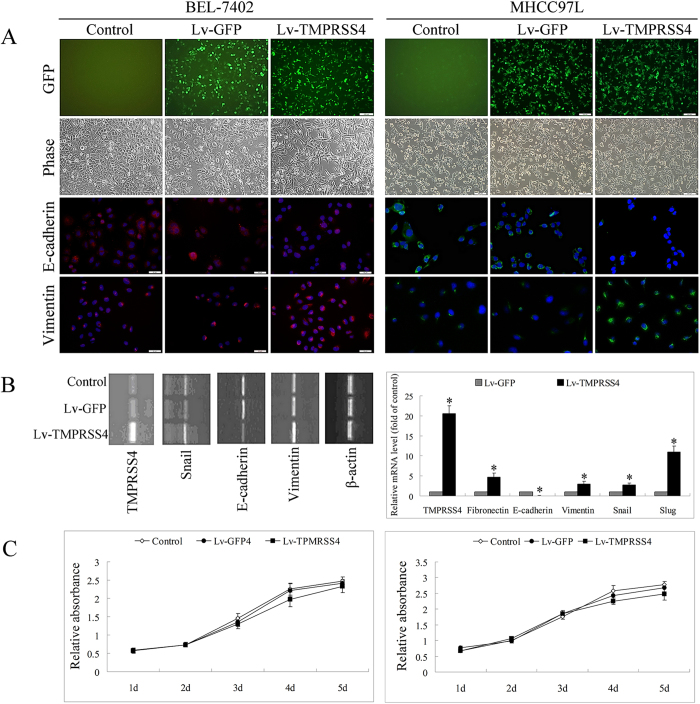
**A** After infection with lentiviruses, over 80% of the transduced cells (Lv-GFP group and Lv-TMPRSS4 group) showed green fluorescence under the fluorescence microscope. Cells transfected with TMPRSS4 altered from an epithelial cobblestone appearance to an elongated/irregular shape, together with enhanced expression of vimentin and reduced expression of E-cadherin in immunofluorescence assay, suggesting an epithelial-mesenchymal transition of TMPRSS4 transfected cells; **B** PCR revealed that cells transfected with TMPRSS4 showed significantly enhanced expression of TMPRSS4, vimentin, snail and slug, but reduced expression of E-cadherin; **C** TMPRSS4 transfection slightly inhibited the *in vitro* proliferation of HCC cells. Bars, SD. *P < 0.05.

Three days after infection with lentiviruses, over 80% of the transduced cells (Lv-GFP group and Lv-TMPRSS4 group) showed green fluorescence under the fluorescence microscope (Fig. 1A). During the cell culture and passaging, the green fluorescence protein was expressed persistently and lasted at least 6 months.

Comparison of the morphology of the control cells, the Lv-GFP transfected cells and Lv-TMPRSS4 transfected cells revealed that cells transfected with TMPRSS4 altered from an epithelial cobblestone appearance to an elongated/irregular shape, suggesting an epithelial-mesenchymal transition. (Fig. 1A). Consistent with the morphology changes of EMT, immunofluorescence assay of HCC cells also revealed marked enhancement of vimentin expression and inhibition of E-cadherin expression after TMPRSS4 transfection (Fig. 1A).

48 hours posttransfection, RNA of EMT related genes were extracted and subjected to RT-PCR or quantitative RT-PCR. Cells transfected with TMPRSS4 showed significantly enhanced expression of TMPRSS4, vimentin, snail and slug, but showed reduced expression of E-cadherin (Fig. 1B).

### TMPRSS4 overexpression promoted the invasion, adhesion and migration of HCC

**Figure 2:**
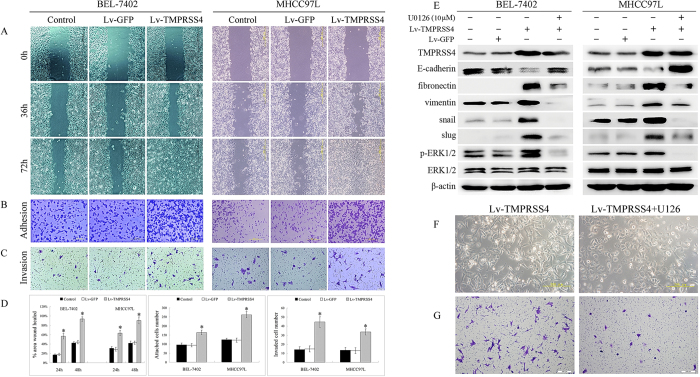
**A** Closing of the wound was much faster in Lv-TMPRSS4 group than in control and Lv-GFP group; **B,C** The number of invading or attached cells increased several-fold over control and vector-transfected cells when TMPRSS4 was overexpressed; **D** Statistical analyses confirmed that cells transfected with TMPRSS4 showed significantly enhanced ability to migration, adhesion and invasion; **E** Compared with control and Lv-GFP group, TMPRSS4-overexpression cells displayed significantly enhanced activation of ERK1/2, together with reduced expression of E-cadherin and enhanced expression of fibronectin, vimentin, snail and slug, which can be significantly reversed by U0126, the inhibitor of ERK1/2; **F** After U0126 treatment, the morphologies of TMPRSS4 overexpression cells were altered from an elongated/irregular shape to an epithelial cobblestone appearance; **G**U0126 treatment resulted in significant suppression of cell invasion. Bars, SD. *P < 0.05.

To examine the effect of TMPRSS4 expression on the development of malignant characteristics in HCC cells, the invasion, adhesion and migration of HCC cells were evaluated. TMPRSS4 overexpression slightly inhibited the *in vitro* proliferation of HCC cells (Fig. 1C), but promoted the migration of HCC cells. Closing of the wound was much faster in Lv-TMPRSS4 group than in control and Lv-GFP group (Fig. 2A), and the number of invading or attached cells of Lv-TMPRSS4 group was also increased several-fold over control and Lv-GFP group (Fig. 2B–D), indicating that the invasive activity of HCC cells was correlated with TMPRSS4 expression level.

### TMPRSS4 overexpression influenced the expression of EMT related genes

Western blot analysis revealed that TMPRSS4 overexpression significantly inhibited the expression of E-cadherin and enhanced the expression of fibronectin, vimentin, snail and slug (Fig. 2E). To further clarify the role of TMPRSS4 in EMT, TMPRSS4 siRNA was developed. After siRNA transfection, substantial knockdown of TMPRSS4 expression was observed in TMPRSS4 overexpression BEL-7402 cells, followed by enhanced expression of E-cadherin and reduced expression of fibronectin, vimentin, pERK1/2, snail and slug (Supplementary Fig. 1).

### TMPRSS4 overexpression induced EMT via Raf/MEK/ERK1/2 pathway

To determine which signaling pathway is related to TMPRSS4 function, phosphorylation of the signaling proteins extracellular signal-regulated kinase (ERK)1/2 of HCC cells was assessed. Compared with control and Lv-GFP cells, TMPRSS4-overexpression cells displayed significantly enhanced activation of ERK1/2 (Fig. 2E). In addition, MEK and C-Raf, the upstream activators of ERK signaling pathway, were also activated by TMPRSS4 (Supplementary Fig. 2). U0126, the inhibitor of ERK1/2, not only inhibited EMT markers of control cell (Supplementary Fig. 3), but significantly inhibited the downregulation of E-cadherin and upregulation of fibronectin, vimentin, snail and slug induced by overexpression of TMPRSS4 (Fig. 2E), indicating that TMPRSS4 induced EMT was mediated through activating Raf/MEK/ERK1/2 pathway. Other signaling pathways, such as PI3K/Akt and signal transducers and activators of transcription 3 (STAT3), were not significantly activated by TMPRSS4 expression, and specific PI3K inhibitor LY294002 (30 μM) did not influence the TMPRSS4 induced downregalation of E-cadherin and upregulation of vimentin (Supplementary Fig. 4 and 5).

### Inhibition of ERK1/2 suppressed TMPRSS4 induced EMT and invasion

**Figure 3:**
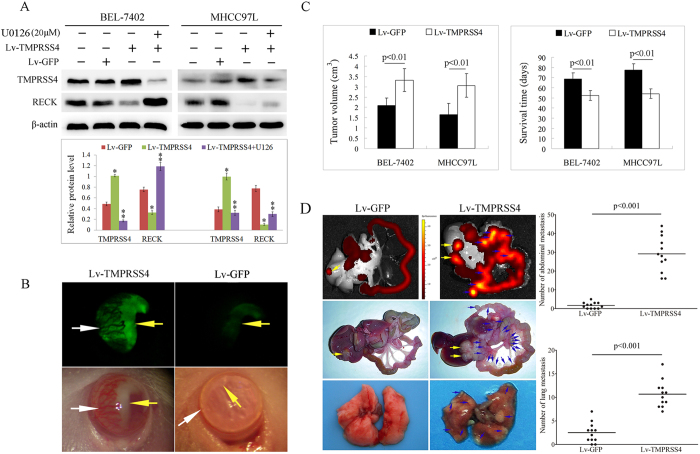
**A** Western blot analysis revealed that cells transfected with TMPRSS4 showed significantly reduced expression of RECK, and ERK1/2 inhibitor of U0126 reversed the effect of TMPRSS4 on RECK expression; **B** TMPRSS4 overexpression HCC cells formed much bigger tumors in the anterior chamber of mice (yellow arrow) with large amounts of angiogenesis induced (white arrow); **C**The tumors of the Lv-TMPRSS4 group were significantly bigger than those of the Lv-GFP group (p < 0.01), and the survival time of Lv-TMPRSS4 group was significantly longer than that of Lv-GFP group (p < 0.01); **D** The mice bearing Lv-GFP tumors showed small luminescence in the liver (yellow arrow) and no luminescence in the peritoneum, whereas mice bearing Lv-TMPRSS4 tumors demonstrated strong luminescence both in the liver (yellow arrow) and in the peritoneum (blue arrow); Pathological examination also revealed that both abdominal and lung metastasis (blue arrow) were significantly higher in Lv-TMPRSS4 group than in Lv-GFP group (p < 0.001). Bars, SD.

To further clarify the role of ERK1/2 in TMPRSS4 induced EMT and invasiveness, we investigated the role of U0126 on the morphologies and invasion of TMPRSS4 overexpression BEL-7402 cells. Consistent with the changes of EMT markers, after U0126 treatment, the morphologies of TMPRSS4 overexpression BEL-7402 cells altered from an elongated/irregular shape to an epithelial cobblestone appearance (Fig. 2F), and U0126 treatment also resulted in significant suppression of cell invasion compared with untreated cells (Fig. 2G). In addition, when the concentration of U0126 increased, inhibition of ERK1/2 not only inhibited the TMPRSS4 induced EMT, but also inhibited the expression of TMPRSS4, indicating that ERK1/2 also provides a feedback regulation for TMPRSS4 expression (Fig. 3A).

### TMPRSS4 overexpression suppressed RECK expression and promoted tumor-induced angiogenesis

Western blot analysis revealed that cells transfected with TMPRSS4 showed significantly reduced expression of RECK, and ERK1/2 inhibitor of U126 reversed the effect of TMPRSS4 on RECK expression, suggesting that TMPRSS4 inhibited the expression of RECK through activating ERK1/2 (Fig. 3A). Since RECK is an inhibitor of angiogenesis, the angiogenesis was further investigated. We found TMPRSS4-overexpression HCC cells formed much bigger tumors in the anterior chamber of mice (yellow arrow, Fig. 3B) with large amounts of angiogenesis induced (white arrow, Fig. 3B), which could contribute to tumor growth.

### Overexpression of TMPRSS4 promoted metastasis of HCC cells *in vivo*

**Figure 4:**
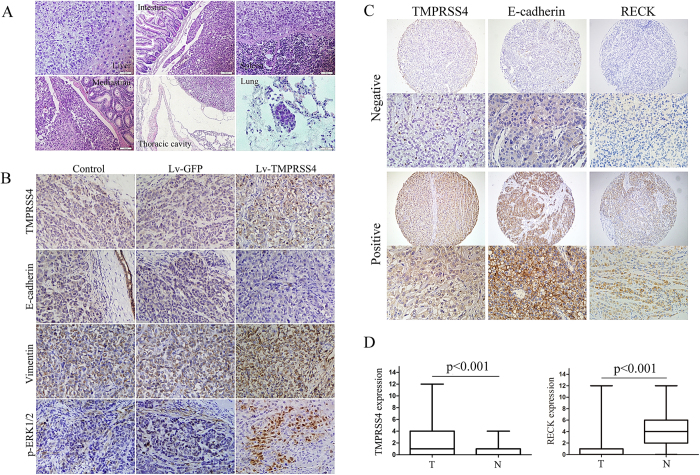
**A** HE stain of TMPRSS4 overexpression tumors that metastasize to live, intestinal mesentery, spleen, mediastinu, thoracic cavity and lung; **B** Immunohistochemical assay of tumors in mice of the control group, Lv-GFP group and Lv-TMPRSS4 group revealed that E-cadherin expression was significantly reduced while the expression of vimentin and activity of ERK1/2 was greatly enhanced in tumors overexpressing TMPRSS4; **C** Positive and negative expression of TMPRSS4, E-cadherin and RECK in human HCC samples by using human tissue microarray; **D**Box-and-whisker plots of the staining. The immunoreactive score of human HCC (T) and adjacent nontumor tissues (N) is shown as mean ± SD.

We next determined whether TMPRSS4 influenced the behavior of tumors *in vivo*. Five weeks after orthotopic inoculation of Lv-GFP or Lv-TMPRSS4 tumor tissue into the liver, some of the mice were sacrificed and metastases were evaluated by luminescence imaging system and pathological examination, the other mice were left for evaluation of survival time. As shown in Fig. 3C, the tumors of the Lv-TMPRSS4 group were significantly bigger than those of the Lv-GFP group (p < 0.01), and the survival time of mice in Lv-TMPRSS4 group was significantly shorter than that of Lv-GFP group (p < 0.01). The mice bearing Lv-GFP tumors showed small luminescence in the liver (yellow arrow, Fig. 3D) and no luminescence in the peritoneum, whereas mice bearing Lv-TMPRSS4 tumors demonstrated strong luminescence both in the liver (yellow arrow, Fig. 3D) and in the peritoneum (blue arrow, Fig. 3D), indicating that TMPRSS4 significantly promoted the metastasis of HCC tumors. Pathological examination also revealed that both abdominal and lung metastasis (blue arrow) were significantly higher in Lv-TMPRSS4 group than in Lv-GFP group (Fig. 3D, p < 0.001). The liver tumor and metastatic nodules were confirmed histologically, and mice bearing Lv-TMPRSS4 tumors exhibited increased metastasis of HCC into various organs such as intestinal mesentery, spleen, mediastinu, thoracic cavity and lung (Fig. 4A). Taken together, these results demonstrated that TMPRSS4 played a key role in driving metastasis of HCC *in vivo*.

### Immunohistochemical staining for EMT-related markers in HCC xenografts of nude mice

Consistent with the changes of EMT markers *in vitro* study, immunohistochemical assay of tumors in mice of the control group, Lv-GFP group and Lv-TMPRSS4 group revealed that E-cadherin expression was significantly reduced while the expression of vimentin and activity of ERK1/2 was greatly enhanced in tumors overexpressing TMPRSS4 (Fig. 4B).

### TMPRSS4 expression is inversely correlated with E-cadherin and RECK expression in primary human HCCs

To further investigate whether TMPRSS4 expression is relevant to human HCC development, we examined the expression of TMPRSS4, E-cadherin and RECK in human HCC samples by using human tissue microarray (Fig. 4C), and tested the association of TMPRSS4 to E-cadherin and RECK expression. We found that TMPRSS4 expression was markedly enhanced in HCC tumors compared to corresponding adjacent nontumor tissues (p < 0.001, Fig. 4D), and positive staining of TMPRSS4 (score, 4–12) was detected in approximately 32.2% (128 of 398) of HCC samples. In contrast, RECK expression was markedly reduced in HCC tumors compared to corresponding adjacent nontumor tissues (Fig. 4D), and RECK staining was positive in approximately 34.7% (138 of 398) of nontumor sections and in 12.3% (49 of 398) of tumor areas.

**Figure 5:**
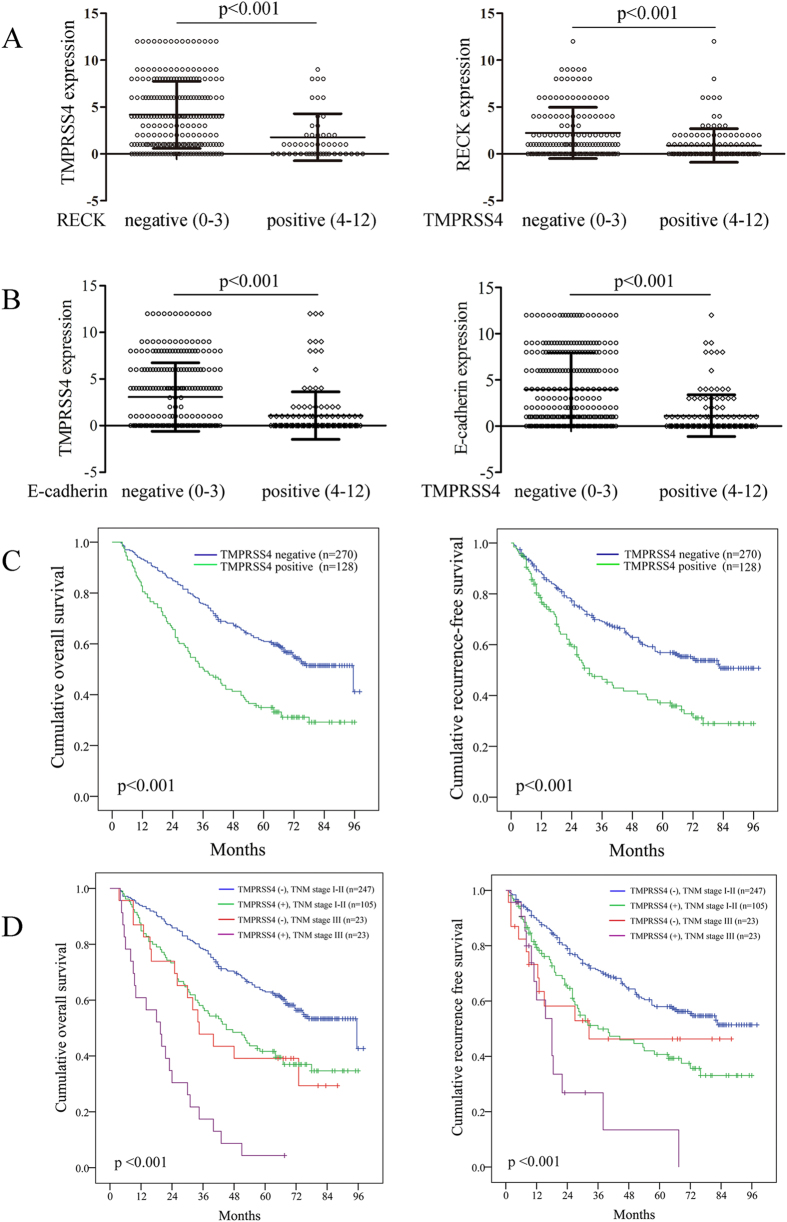
**A** Statistical analysis of TMPRSS4/RECK expression in HCC sections negative (score, 0–3) and positive (score, 4–12) for RECK/TMPRSS4 staining (p < 0.001); **B** Statistical analysis of TMPRSS4/E-cadherin expression in HCC sections negative and positive for E-cadherin/TMPRSS4 staining (p < 0.001). The immunoreactive score is shown as mean ± SD; **C** The prognostic significance assessed by using Kaplan-Meier survival estimates and log-rank tests. Comparisons of OS and DFS by TMPRSS4; **D** Comparisons of OS and DFS by TMPRSS4 and TNM stage.

Statistical analysis showed a significant difference in TMPRSS4 expression in HCC regions between RECK negative and positive groups (Fig. 5A). When categorized with TMPRSS4 expression in HCC sections, the positive group had significantly lower RECK expression than the negative group (Fig. 5A, p < 0.001). In addition, a significant difference in TMPRSS4 expression in HCC regions between E-cadherin negative and positive groups was detected (Fig. 5B). When categorized with TMPRSS4 expression in HCC sections, the positive group had significantly lower E-cadherin expression than the negative group (Fig. 5B, p < 0.001). These results indicate that TMPRSS4 expression is inversely correlated with E-cadherin and RECK expression in human HCC.

### Expression of TMPRSS4 is predictive of poor prognosis in HCC patients after curative resection

In this study, despite the risk factors such as vascular invasion and tumor number, multivariate analysis reveled that TMPRSS4 expression was an independent risk factor for both OS (p < 0.001, HR: 2.20, 95% CI: 1.66–2.90) and recurrence (p < 0.001, HR: 1.95, 95% CI: 1.45–2.63) after curative resection (Table 1). The 1-, 3-, and 5-year OS rates after curative resection for TMPRSS4 positive patients were 82.2%, 50.0% and 34.9%, significantly worse than those of TMPRSS4 negative patients (93.3%,75.6% and 60.9%, p < 0.001, Fig. 5C). The 1-, 3-, and 5-year RFS rates of TMPRSS4 positive patients were also significantly worse than those of TMPRSS4 negative patients (76.8%, 46.4% and 37.1% vs. 87.9%, 69.0% and 56.9%, p < 0.001, Fig. 5C).

When we further stratified patients by TNM stage, we found that most of the patients in TNM stage I and II were TMPRSS4 negative (247 of 352), while half of the patients in TNM stage III were TMPRSS4 positive (p = 0.006, Table 2), suggesting the expression of TMPRSS4 was significantly correlated with tumor staging. The 5-year OS and DFS rates of TMPRSS4 negative patients in TNM stage I and II were significantly better compared with TMPRSS4 positive patients in the same stage (62.9% vs. 41.7%, p < 0.001; 58.0% vs. 40.7%, p < 0.001, respectively; Fig. 5D). The survival of TMPRSS4 negative patients in TNM stage III were also significantly better than TMPRSS4 positive patients in the same stage (p < 0.001, Fig. 5D), and nearly all TMPRSS4 positive patients in TNM stage III (22 of 23) died within 5 years.

### Comparison of clinicopathologic characteristics of TMPRSS4 positive and TMPRSS4 negative patients

The clinicopathologic characteristics of TMPRSS4 positive and TMPRSS4 negative patients were compared, and Table 3 showed the baseline demographic data and tumor characteristics of them. Though there were no significant differences between these two groups regarding gender, age, HBsAg status, AFP, GGT and ALT level, cirrhosis, tumor capsule, tumor number and tumor differentiation, tumor size was significantly larger in TMPRSS4 positive group than in negative group (p = 0.021). Tumors of TMPRSS4 positive group had a higher incidence of vascular invasion (p = 0.043), and a higher incidence of postoperative recurrence than those of TMPRSS4 negative group (p = 0.011).

### Comparison of clinical data of recurrent patients with positive or negative TMPRSS4 expression

The clinical data of recurrent patients are summarized in Table 4. Among the 184 recurrent patients, 71 patients were TMPRSS4 positive while the other 113 patients were TMPRSS4 negative. All these recurrent cases were divided into early or late-recurrence groups, using 1 year as the cutoff value. The median survival time of TMPRSS4 positive patients was 36.5 months, significantly shorter than that of TMPRSS4 negative patients, and the 5-year OS of TMPRSS4 positive recurrent patients were significantly worse than those of TMPRSS4 negative recurrent patients (24.6% vs 42.3% p = 0.011). TMPRSS4 positive patients tends to recur earlier than TMPRSS4 negative patients (18 mos vs 23.5 mos, p = 0.109), and the majority (65%) of TMPRSS positive recurrence were early recurrence while the majority (72%) of TMPRSS4 negative recurrence were late recurrence (p < 0.001). In addition, the incidence of extrahepatic recurrence was higher in TMPRSS4 positive group than in TMPRSS4 negative group (p = 0.033).

## Discussion

The role of TMPRSS4 on invasion and migration of other types of cancer cells has been established^1^,^10^. In the present study, we investigated the role of TMPRSS4 on HCC growth and metastasis *in vivo* and *in vitro*. Our results showed that TMPRSS4 significantly enhanced the invasion, migration and adhesion of HCC cells by several folds, but did not promote the *in vitro* proliferation of HCC cells. It’s reported that TMPRSS4 modulates cell growth in a cell type-dependent manner and it can promote the proliferation of certain types of cells by regulating cell cycle progression, but does not confer a growth advantage to other types of cells^1^, just as we found in this study. In addition, TMPRSS4 significantly upregulated the mesenchymal markers and inhibited the expression of epithelial markers of HCC cells, together with morphological changes that are characteristic of motile fibroblasts, indicating that TMPRSS4 induces EMT of HCC cells.

EMT is a process implicated in the conversion of early stage tumors to invasive malignancies. Induction of EMT will result in weakened intercellular adhesion and enhanced cell motility, thereby allow tumor cells to metastasize and establish secondary tumors at distant site^11^,^12^,^13^,^14^. Snail-related zinc-finger transcription factors such as snail and slug are the most important EMT-activating transcription factors^15^,^16^,^17^, which are regulated either directly or indirectly by a series of intracellular signaling networks^18^. In this study, we observed strong snail and slug induction, as well as Raf/MEK/ERK1/2 activation, in TMPRSS4-overexpressing transfectants, while ERK1/2 inhibitor significantly diminished the TMPRSS4 induced down-regulation of E-cadherin and up-regulation of vimentin, snail, and slug, suggesting that TMPRSS4 induced EMT was mediated through snail and slug as a result of Raf/MEK/ERK1/2 activation. Other signaling pathways, such as PI3K/Akt and STAT3, were not significantly activated by TMPRSS4 expression, and were not involved in TMPRSS4 induced EMT.

More over, we found that overexpression of TMPRSS4 also significantly suppressed the expression of RECK, together with increased tumor angiogenesis, through activating ERK1/2 pathway. Though reduced level of TMPRSS4 has been proved to cause severe defects in embryonic development including a degenerated vascular system^19^, TMPRSS4 or any other TTSP family has never been linked to cancer angiogenesis in previous studies. RECK is expressed in various of human tissues and untransformed cells, and it is undetectable in tumor-derived cell lines and oncogenically transformed cells^20^. As an inhibitor of angiogenesis^21^,^22^, forced expression of RECK in cancer cells results in reduced angiogenesis and suppression of MMPs, thereby suggesting effective applications of RECK in cancer therapy^21^,^23^. Intriguingly, including ERK, several signaling pathways, which have been suggested to be involved in the regulation of RECK expression^24^,^25^,^26^, are also known to induce EMT. Therefore, a link between angiogenesis, RECK and EMT may exist for their common regulatory pathways^27^, and the role of RECK in TMPRSS4 induced EMT is interesting subjects to be explored in future studies.

As HCC tumors have the tendency to metastasize to lung and peritoneal organs^28^, peritoneal dissemination and lung metastasis of orthotopically implanted HCC tumors in nude mice were used as parameters to measure HCC metastasis. As compared with control tumors, TMPRSS4 overexpression tumors showed significantly reduced E-cadherin expression, enhanced expression of vimentin and activation of ERK1/2, as well as increased extrahepatic metastasis such as peritoneal dissemination and lung metastasis. Though expression of TMPRSS4 did not promote the proliferation of HCC cells in our *in vitro* study, however, tumors of TMPRSS4 overexpression group were significantly larger than that of control group. The *in vivo* angiogenesis induced by TMPRSS4 via inhibiting RECK expression may account for the discrepancy between the *in vivo* and *in vitro* studies. In agreement with these animal studies, our clinical analysis also revealed that TMPRSS4 levels in HCC were inversely correlated with RECK and E-cadherin expression, and patients of TMPRSS4 positive group had a higher incidence of vascular invasion and tumors were much lager than those of TMPRSS4 negative group. All these results indicate that TMPRSS4 was an important indicator for progressive HCC.

TMPRSS4 expression is upregulated in malignant tumors compared to benign neoplasm or normal tissues, and therefore is suggested as both a diagnostic and prognostic marker for cancers^29^. In this study, TMPRSS4 was positive in approximately 32% of HCC tumors, significantly higher than the adjacent nontumor regions. Although not all the tumors expressed TMPRSS4, the expression of TMPRSS4 predicted very poor prognosis regardless of AFP level, tumor size, tumor number, and vascular invasion-the putative prognostic factors in HCC^30^,^31^,^32^. In addition, positive TMPRSS4 expression was also an independent risk factor for postoperative recurrence, and the recurrence rates, especially extrahepatic recurrence rates, were significantly higher in TMPRSS4 positive group than in TMPRSS4 negative group. More importantly, positive TMPRSS4 expression was also predictive of early recurrence of HCC in this study, and early recurrence rate was significantly higher in TMPRSS4 positive patients than in TMPRSS4 negative patients. Therefore, the predictive value of TMPRSS4 is very valuable to help clinicians to distinguish high risk recurrence, and patients with positive TMPRSS4 expression should be closely monitored for early detection of recurrence and extrahepatic metastasis, thereby take preventive and therapeutic measures for better prognosis.

In addition, though TMPRSS4 expression in HCC was significantly correlated with tumor staging, the survival rates of TMPRSS4 positive patients in early stage (TNM stage I-II) were still significantly reduced compared with TMPRSS4 negative patients in the same stage, suggesting that TMPRSS4 may also be a promising marker for poor prognosis in early stage HCC patients.

In summary, this study provides novel insight into a function of TMPRSS4 in activating the ERK1/2 pathway and promoting invasion and metastasis of HCC through induction of EMT. The increase of TMPRSS4 expression is a key event for HCC progression and may be regarded as a potential prognostic marker for HCC. The present findings also identify link between TMPRSS4 and RECK that may enhance our understanding of tumor angiogenesis and lead to the development of novel treatment strategies.

## Methods

The experiments were approved by the Ethic Committee of Zhongshan Hospital, Fudan University, performed in accordance with the approved guidelines, and informed consent was obtained from all subjects.

### Cell line and animal model

Two commonly used human HCC cell line MHCC97L and BEL-7402 were used in this study. MHCC97L cell line was established at our institute (Liver cancer institute of Fudan University). It was established from subcutaneous xenograft of a metastatic model of human HCC in nude mice by means of alternating cell culture *in vitro* and growth in nude mice. The cells, either grown as compact colonies or as a monolayered sheet, exhibited typical malignant epithelia in morphology. The cell line was shown to be of human origin by karyotype analysis and retain some properties of the original tumor, including AFP synthesis and metastatic potential^33^. BEL-7402 cell line was obtained from the Cell Bank of the Shanghai Institute of Materia Medica, Chinese Academy of Sciences. This cell line was derived from liver carcinoma specimens of operated patients and has been successfully established *in vitro*^34^. The cells appeared as epithelial-like cells in morphology and showed, in addition to desmosomes, the presence of cytoplasmic tonofibrills which were typical of epithelial cells by electron microscopy. The cells had hypotriploid chromosone number and AFP was detected intracellularly. BEL-7402 also had metastatic potential. Cells were maintained in DMEM, supplemented with 10% fetal bovine serum and 1% streptomycin/penicillin at 37 °C with humidified 95% air and 5% CO2.

Male BALB/c nu/nu mice, 4–6 weeks old, were obtained from Shanghai Institute of Materia Medica, Chinese Academy of Science. All studies on mice were conducted in accordance with the National Institutes of Health “Guide for the Care and Use of Laboratory Animals” and were approved by Shanghai Medical Experimental Animal Care Committee. Animal model was established by orthotopic inoculation of histologically intact tumor tissue (2 × 2 × 2 mm) into the liver^35^. Each group contained 12 to 18 mice.

2 μL of 1.0 × l0^6^ mL BEL-7402 cells was injected into the anterior chamber in one eye of each Balb/c mice as we previously described^36^. The neoplasms in anterior chamber were observed under the fluorescence microscope on the 15, 30 and 45 days postoperatively to investigate tumor growth and tumor-induced angiogenesis

### Cell Growth Assay

The proliferation of cells was evaluated by the MTT assay. Cells were plated in a 96-well plate at 2.5 × 10^3^ cells/well and were allowed to grow for different times. The growth rate was determined by the cell number and was counted in triplicate every day by MTT assay. Briefly, cells were incubated with 50 μL of 0.2% MTT for 4 h at 37 °C in a 5% CO_2_ incubator. Following MTT incubation, 150 μL of 100% DMSO was added to dissolve the crystals. Viable cells were counted every day by reading the absorbance at 490 nm using a 96-plate reader BP800 (Dynex Technologies).

### Cell invasion, adhesion and migration assay

Invasiveness of MHCC97L and BEL-7402 cells were measured by the invasion of cells through Matrigel coated transwell inserts (Corning, USA). Briefly, transwell inserts with 8 μm pores were coated with Matrigel (40 μg/well; Becton Dickinson, Bedford, USA). 1 × 10^5^ cells were added to the upper chamber, suspended in 100 μl of DMEM. The lower chamber was filled with 600 μl of DMEM supplemented with 0.1% BSA. After 36 h incubation, the cells on the upper surface were removed. The remaining invaded cells were counted at ×200 magnifications in 5 different fields of each filter.

96-well plates coated with 10 μg/mL fibronectin (Calbiochem, San Diego, CA) were used for adhesion assay. After a 30-min incubation at 37 °C, adherent cells were fixed and stained. Wound healing assay was used to detect cell migration. Cells were seeded onto 6-well plate (1.0 × 10^5^ per well). When cells were grown to 70% confluence, an artificial wound was carefully created using P200 pipette tip to scratch on the confluent cell monolayer. Microphotographs were taken at 0 h, 36 h and 72 h post wounding.

### Lentivirus Infection

5 × 10^5^ cells per well were seeded in 6-well plates the day before transfection. The next day, pGC-FU-TMPRSS4-GFP-lentivirus and pGC-FU-GFP-lentivirus (Shanghai GeneChem, China) were added respectively into HCC cells with 1 ml fresh DMEM/F12 containing 10% FBS, 5 μg/ml Polybrene (Sigma, USA). Twelve hours later, the medium was removed and replaced with fresh culture medium. Three days later, the GFP gene expression was observed under the fluorescence microscope and the cells were collected for subsequent culture. Expressions of TMPRSS4 or TMPRSS4 mRNA were analyzed by western blotting or RT-PCR.

### siRNA design and assay

BEL-7402 cells were incubated without antibiotics for 24 h before transfection. Control siRNA labeled with FAM and specific TMPRSS4 siRNA were mixed with Lipofectamine2000 (Invitrogen) according to the manufacturer’s recommendation and added to the cells. After 6 h at 37 °C, the medium was changed and the cells were cultured in RPMI 1640 supplemented with 10% fetal bovine serum. Transfection efficiency was observed under a fluorescence microscope. The silencing level of TMPRSS4 was determined by western blot 48 h after transfection. To minimize the off-target effect of RNAi, three TMPRSS4 siRNAs that have been demonstrated capable of suppressing TMPRSS4 expression were mixed together as a pool that was named siRNA-TMPRSS4. The sequences for the three siRNAs were siRNA-1, sense 5′-GGAUCCUGACAGUGAUCAATT-3′ and antisense 5′-UUGAUCACUGUCAGGAUCCTG-3′; siRNA-2, sense 5′-CCGAUGUGUUCAACUGGAATT-3′ and antisense 5′-UUCCAGUUGAACACAUCGGTA-3′; siRNA-3, sense 5′-AGGUGAUUCUGGAUAAAUATT-3′ and antisense 5′-UAUUUAUCCAGAAUCACCUTG-3′; FAM-labeled siRNA was used as a control (sense 5′-UUCUCCGAACGUGUCACG-3′ and antisense 5′-ACGUGACACGU UCGGAGA-3′). The siRNAs were chemically synthesized at the Laboratory of RNA Chemistry (Shanghai GenePharma, Shanghai, China).

### Western blot analysis

For Western blot analysis, 50 μg of total protein lysates (RIPA lysis buffer) were subjected to SDS-PAGE. The membrane was incubated with monoclonal anti-human TMPRSS4, E-cadherin, vimentin, fibronectin (1:1000, Santa Cruz Biotechnology, USA), Slug, Snail, ERK1/2, pERK1/2, pAkt, pMEK, STAT3 (1:1000, Cell Signaling Technology, USA). Then probed with anti-mouse/rabbit IgG (Cell Signaling Technology, USA) at 1:10000 dilution for 1 h.

### RNA isolation and quantitative RT-PCR

Total RNA was extracted from tumors using Trizol reagent (Invitrogen, Carlsbad, CA). cDNA was synthesized using the SuperScript first strand synthesis system (Invitrogen). Portions of double-stranded cDNA were subjected to PCR with a SYBR Green PCR kit (Applied Biosystems, Foster City, CA, USA). The amplification protocol comprised incubations at 94 °C for 15 s, 63 °C for 30 s, and 72 °C for 60 s. Incorporation of the SYBR Green dye into PCR products was monitored in real time using an ABI Prism 7700 sequence detection system (PE Applied Biosystems, Foster City, CA, USA), thereby allowing determination of the threshold cycle (CT) at which exponential amplification of the products began. The target cDNA was then amplified by PCR. Primers were described in Supplementary Table 1.

### Immunofluorescence Assay

E-cadherin and Vimentin expression in the control cells, the Lv-GFP transfected cells and Lv-TMPRSS4 transfected cells was detected by immunofluorescence assay. Briefly, cells cultured on glass slides were fixed by acetone for 15 min. After treated with 0.2% Triton-X-100 for 2 min, the fixed cells were blocked with bovine serum albumin and stained with FITC conjugated monoclonal antibody (1:200, Santa Cruz Biotechnology, USA) for 1 h at 37 °C. After rinsing in PBS, the slices were counterstained with DAPI (Vector Laboratories, Inc. Burlingame, USA) and examined under fluorescent microscope (Olympus BX-40, Japan).

### Patients and Specimens

Archival specimens used for tissue microarray (TMA) were obtained from 398 patients at Liver Cancer Institute, Zhongshan Hospital, Fudan University between 2006 and 2008. TMA were constructed by Shanghai Outdo Biotech Company. The inclusion and exclusion criteria of the patient cohorts include (a) having a distinctive pathologic diagnosis of HCC, (b) having no anticancer treatment before liver resection, (c) having curative liver resection, (d) having suitable formalin-fixed, paraffin-embedded tissues or frozen tissues, and (e) having a complete clinicopathologic and follow-up data. The patient information was listed in Table 3. Ethical approval for human subjects was obtained from the research ethics committee of Zhongshan Hospital; informed consent was obtained from each patient.

Curative resection was defined as complete resection of all tumor nodules and the cut surface being free of cancer by histologic examination; having no cancerous thrombus in the portal vein (main trunk or two major branches), hepatic veins, or bile duct; and having no extrahepatic metastasis^37^. The staging of tumors was determined according to the TNM classification system of the 7th edition. The histological grade of tumor differentiation was assigned by the Edmondson grading system^38^.

### Follow-Up

All the 398 patients were followed up and the median follow-up time was 63 months (range, 2–98 months). Patients were followed regularly in the outpatient clinic and were monitored prospectively for recurrence according to a standard protocol as previously described^30^. All patients were monitored prospectively by serum AFP, liver function, ultrasonography and chest X-ray every two months, and contrast enhanced computed tomography (CT) was performed every 6 months. Bone scan or magnetic resonance imaging (MRI) was performed if localized bone pain was reported. A diagnosis of recurrence was based on typical imaging appearance in CT and/or MRI scan and an elevated AFP level. Recurrences were divided into early and late recurrences using 1 year as the cutoff value, as suggested by previous study^39^.

### Evaluation of Immunohistochemical Variables

The TMA sections were used for immunochemistry staining. Monoclonal antibodies against human TMPRSS4 (1:100), E-cadherin (1:50) were purchased from DakoCytomation, Denmark. Immunohistochemistry was carried out using a two-step protocol (Novolink Polymer Detection System, Novocastra, Newcastle, UK) as previously described^8^. Briefly, after microwave antigen retrieval, tissues were incubated with primary antibodies for 60 min at room temperature. Following 30 min incubation with secondary antibody, the sections were developed in DAB solution under microscopic observation and counterstained with hematoxylin. Negative control slides with the primary antibodies omitted were included in all assays.

For Immunohistochemistry staining, five fields of approximately 500 cells from each tumor and adjacent nontumor sections were counted independently by 2 pathologists. TMPRSS4, RECK and E-cadherin staining were reported separately according to the German semiquantitative scoring system. Briefly, depending on the percentage of staining intensity, the staining was classified into 4 groups: (no staining = 0; weak staining = 1; moderate staining = 2; and strong staining = 3) and the percentage of stained cells (0% = 0; 1%–25% = 1; 26% 50% = 2; 51%–75% = 3; and 76%–100% = 4). Final immunoreactive scores were determined by the formula: overall scores = intensity score × percentage score. The overall score ≤ 3 was defined as negative, >3 as positive.

### Statistical analyses

The chi-square test or the Fisher exact probability test was used to evaluate categoric variables, and the Student t test was used to evaluate continuous variables. The cumulative overall survival (OS) rate was calculated using the Kaplan-Meier method and was compared using the log-rank test. Overall survival was calculated from the date of resection to the date of death regardless of the cause of death. Recurrence free survival (RFS) rate was calculated from the date of resection to the date when tumor recurrence was diagnosed or from date of the resection to the last visit, if recurrence was not diagnosed, and the patients were censored at the date of death or the date of last follow-up^40^.

Statistical analyses were performed using the SPSS statistical software package (version 13.0; SPSS Inc., Chicago, IL). Two-tailed p values < 0.05 were considered statistically significant.

## Additional Information

**How to cite this article**: Wang, C.-H. *et al.* TMPRSS4 facilitates epithelial-mesenchymal transition of hepatocellular carcinoma and is a predictive marker for poor prognosis of patients after curative resection. *Sci. Rep.* **5**, 12366; doi: 10.1038/srep12366 (2015).

## Supplementary Material

Supplementary Information

## Figures and Tables

**Table 1:** Multivariate analysis of risk factors related to OS and recurrence of HCC patients.

**Variable**	**HR**	**95% CI**	***p***
OS			
AFP, ng/mL			
≤20	1		
>20	1.58	1.17–2.13	.003
Cirrhosis			
no	1		
yes	1.66	1.10–2.49	.016
GGT, U/L			
≤50	1		
>50	1.35	1.01–1.81	.045
Tumor size, cm			
≤5	1		
>5	1.58	1.19–2.09	.001
Vascular invasion			
no	1		
yes	1.66	1.23–2.46	.001
Tumor number			
single	1		
multiple	1.68	1.23–2.30	.001
TMPRSS4 expression			
Negative	1		
Positive	2.20	1.66–2.90	<.001
Recurrence			
GGT, U/L			
≤50	1		
>50	1.53	1.13–2.07	.006
Vascular invasion			
no	1		
yes	1.40	1.00–1.95	.050
Tumor number			
single	1		
multiple	1.89	1.34–2.67	<.001
TMPRSS4 expression			
Negative	1		
Positive	1.95	1.45–2.63	<.001

Overall survival: OS; Hazard Ratio: HR; Confidence Interval: CI; γ—glutamyltransferase: GGT; AFP: a-fetoprotein; TMPRSS4: transmembrane serine protease 4.

**Table 2:** Analysis of TMPRSS4 expression in HCC at different TNM stages.

**TNM staging**	**No. of cases**	**TMPRSS4 expression**		
		**Positive (0**–**3)**	**Negative (4**–**12)**	**Mean**
I-II	352	105	247	2.45
III	46	23	23	3.67
P value		0.006		0.022

TMPRSS4: transmembrane serine protease 4.

**Table 3:** Comparison of patient demographics and clinical characteristics between TMPRSS4 positive and negative patients.

**Variable**	**Number of patients (%)**		***p***
	**TMPRSS4 positive**	**TMPRSS4 negative**	
	**n = 128**	**n = 270**	
Gender (%)			0.111
male	107 (84)	241 (89)	
female	21 (16)	29 (11)	
Age, yrs (%)			0.280
≤50	58 (45)	138 (51)	
>50	70 (55)	132 (49)	
HBsAg (%)			0.607
positive	103 (80)	223 (83)	
negative	25 (20)	47 (17)	
AFP, ng/mL (%)			0.941
≤20	46 (33)	96 (37)	
>20	82 (67)	174 (63)	
ALT, U/L (%)			0.656
≤75	117 (91)	243 (90)	
>75	11 (9)	27 (10)	
GGT, U/L (%)			0.784
≤50	54 (42)	110 (41)	
>50	74 (58)	160 (59)	
Cirrhosis (%)			0.295
yes	108 (84)	216 (80)	
no	20 (16)	54 (20)	
Tumor size, cm (%)			0.021
≤5	63 (49)	166 (61)	
>5	65 (51)	104 (39)	
Tumor number (%)			0.217
single	98 (77)	221 (82)	
multiple	30 (23)	49 (18)	
Tumor capsule (%)			0.131
yes	68 (53)	165 (61)	
no	60 (47)	105 (39)	
Vascular invasion (%)			0.043
yes	41 (31)	60 (22)	
no	87 (69)	210(78)	
Tumor differentiation (%)			0.832
I-II	83 (65)	178 (66)	
III-IV	45 (35)	92 (34)	
Recurrence (%)			0.011
no	57 (45)	157 (58)	
yes	71 (55)	113 (42)	

ALT: alanine aminotransferase; GGT: γ—glutamyltransferase; AFP: a-fetoprotein; TMPRSS4: transmembrane serine protease 4.

**Table 4:** Clinical Data of Recurrent HCC Patients.

**Characteristics**	**TMPRSS4 positive (n = 71)**	**TMPRSS4 negative (n = 113)**	**P**
Age ≤50, yrs (%)	33 (46)	54 (48)	0.775
Time of recurrence (median, mos)	18	23.5	0.109
Overall survival (median, mos)	36.5	50.5	0.011
Recurrence time (%)			<0.001
Early recurrence	46 (65)	32 (28)	
Late recurrence	25 (35)	81 (72)	
Recurrence site (%)			0.033
Intrahepatic	57 (80)	103 (91)	
Extrahepatic	14 (20)	10 (9)	

TMPRSS4: transmembrane serine protease 4.
