# Supplementary material for: TMPRSS4 facilitates epithelial-mesenchymal transition of hepatocellular carcinoma and is a predictive marker for poor prognosis of patients after curative resection
Source: Sci Rep. 2015 Jul 20;5:12366. doi: 10.1038/srep12366 (PMC4507176; doi:10.1038/srep12366)
Supplement: Supplementary Information [file srep12366-s1.pdf]

TMPRSS4 facilitates epithelial-mesenchymal transition of hepatocellular carcinoma and is a predictive marker for poor prognosis of patients after curative resection

Cheng-Hao Wang[1,2 *], Zhong-Yi Guo[1 *], Ze-Ting Chen[1 *], Xu-Ting Zhi[1], Deng-Ke Li[1], Zhao-Ru Dong[2], Zhi-Qiang Chen[1], San-Yuan Hu[1], Tao Li[1]

Supplementary Table 1. Primers used for QRT-PCR

Gene	Forward	Reverse
E-cadherin	CCACCAAAGTCACGCTGAATAC	GGAGTTGGGAAATGTGAGCAA
Vimentin	GGAAGAGAACTTTGCCGTTGAA	GTGACGAGCCATTTCCTCCTT
TMPRSS4	CCGTTACCTATGTTGTCATTG	CTCACTCTTGCTGCCACTCA
Fibronectin	GAGCTATTCCCCTGCACCTGATG	CGTGCAAGGCAACCACACT
Snail	GAGGCGGTGGCAGACTAG	GACACATCGGTCAGACCAG
Slug	ACACACACACACCCACAGAG	AAATGATTTGGCAGCAATGT
GAPDH	AAGGTCGGAGTCAACGGATTTG	CTTGACAAAGTGGTCGTTGAGG
β-actin	CCAACCGCGAGAAGATGA	CCAGAGGCGTACAGGGATAG

Supplementary figures

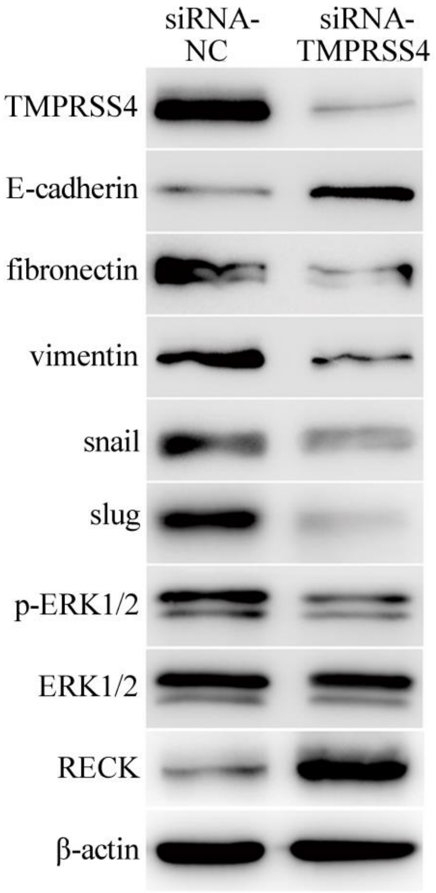

Supplementary Fig 1. After siRNA transfection, substantial knockdown of the TMPRSS4 expression was observed in TMPRSS4 overexpression BEL-7402 cells, followed by enhanced expression of E-cadherin and RECK, and reduced expression of fibronectin, vimentin, pERK1/2, snail and slug.

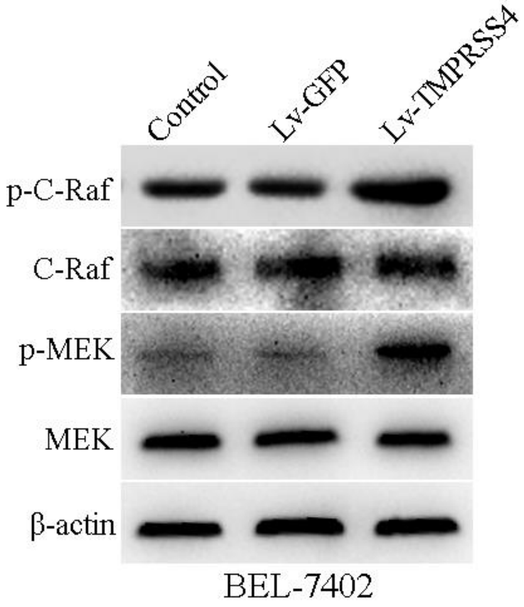

Supplementary Fig 2. Overexpression of TMPRSS4 significantly activated MEK and C-Raf, the upstream activators of ERK signaling pathway.

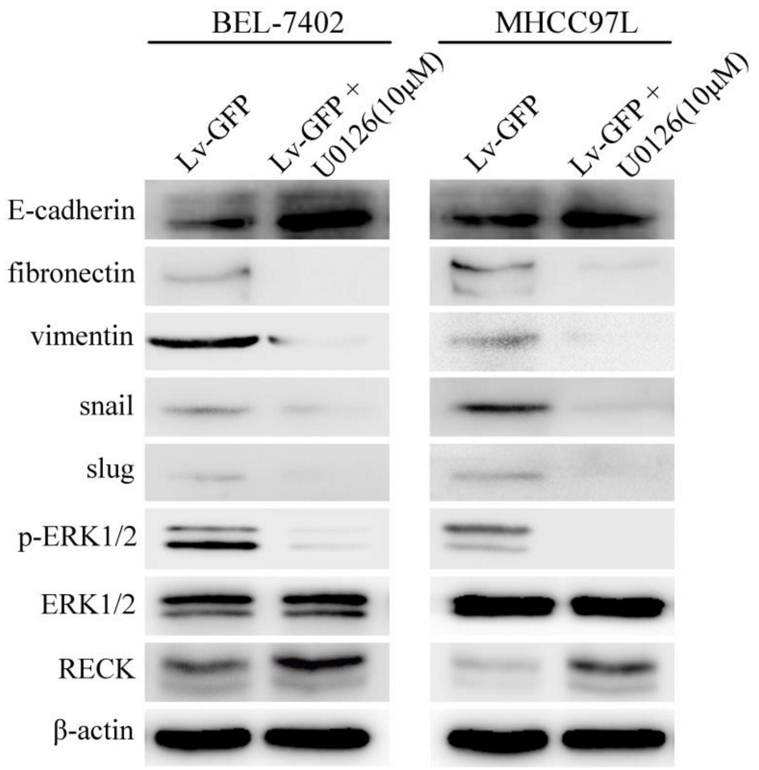

Supplementary Fig 3. U0126, the inhibitor of ERK1/2, enhanced the expression of E-cadherin and RECK, and reduced the expression of fibronectin, vimentin, p-ERK1/2, snail and slug

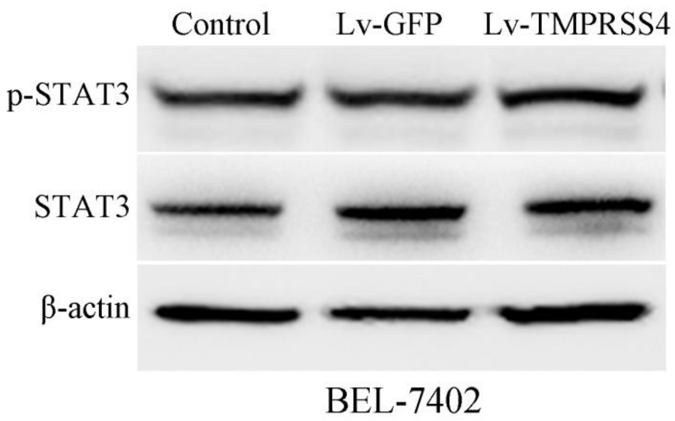

Supplementary Fig 4. Overexpression of TMPRSS4 did not significantly influence the activation of STAT3.

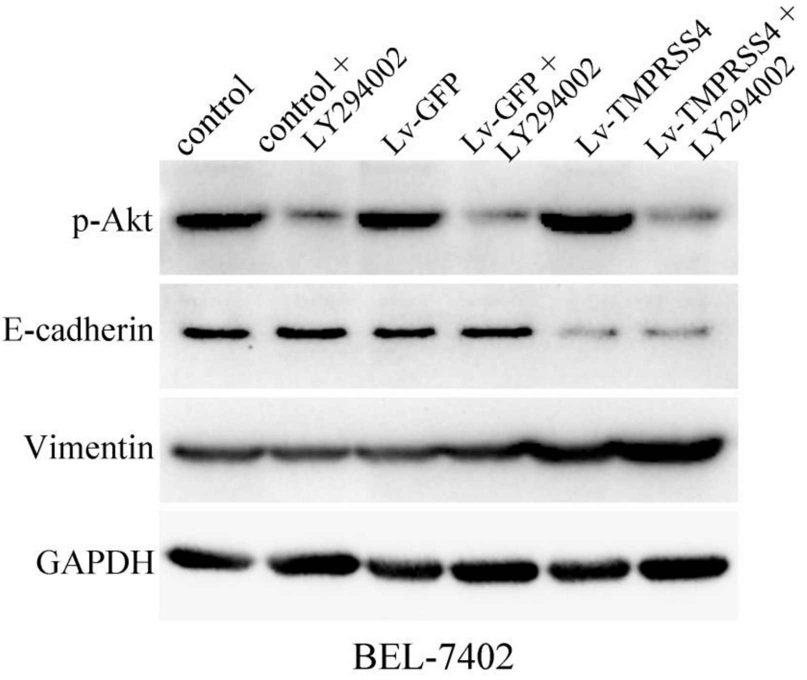

Supplementary Fig 5. Akt was not significantly activated by TMPRSS4 expression, and specific PI3K inhibitor LY294002 (30 μM) did not influence the TMPRSS4 induced downregalation of E-cadherin and upregulation of Vimentin.
